# Personal

**Published:** 1889-01

**Authors:** 


### Personal.

Dr. Louis A. Weigel, of Rochester, announces to his professional acquaintances that he has removed his office to No. 54 North Clinton street. He will continue to give special attention to orthopedic surgery, in which he has achieved such distinction.

---

Mr. Herman A. Hasslock, of Hasslock & Ambrose, our publisher, is a candidate for Public Printer under the incoming administration, and, if promptness, energy, and a thorough knowledge of the art, is a recommendation, he will fill the place with credit to the government and himself. We cordially commend him in every particular, as our relations with him have been close, pleasant, and profitable.—*Nashville Journal of Medicine and Surgery, Dec.*, 1888.

